# Optimization of Growth Conditions and Biological Activities of Nepalese *Ganoderma lucidum* Strain Philippine

**DOI:** 10.1155/2021/4888979

**Published:** 2021-10-04

**Authors:** Krishna Subedi, Buddha Bahadur Basnet, Raju Panday, Manisha Neupane, Giri Raj Tripathi

**Affiliations:** ^1^Central Department of Biotechnology, Tribhuvan University, Kirtipur, Kathmandu, Nepal; ^2^Faculty of Sciences, Nepal Academy of Science and Technology, Khumaltar, Lalitpur, Nepal; ^3^National Forensic Science Laboratory, Khumaltar, Lalitpur, Nepal; ^4^Department of Biotechnology, National Institute of Science and Technology, Lainchaur, Kathmandu, Nepal

## Abstract

*Ganoderma lucidum* has been extensively studied for its valuable medicinal importance. In this study, the artificial cultivation of *G. lucidum* strain Philippine in different culture media, including sawdust substrate, was performed and optimized on the Potato Dextrose Agar (PDA) media. Phytochemical, antibacterial, and antioxidant analyses were performed and compared between the ethanol extracts prepared from two different cultures (fruit from synthetic log culture and mycelia from PDA media culture). Both the 200 mg/mL and 100 mg/mL concentrations of extracts inhibited all the tested bacteria, and the results were promising than the corresponding control using antibiotics. The fruit extract showed higher antioxidant potential (150.6 ± 56.92 mg ascorbic acid equivalent/g extract) than mycelial extract (144.28 ± 81.72 mg ascorbic acid equivalent/g extract). The results indicate that fruiting bodies of *G. lucidum* cultivated in a complex dust medium possess higher antioxidant properties than mycelia culture, which can be further explored for therapeutic applications.

## 1. Introduction

*Ganoderma* (Basidiomycota, Polyporales) is one of the well-known medicinal polypore fungi in the family Ganodermataceae and is widely used in East Asia, America, and other countries [1]. Among the known, more than 400 species, only red, white, black, yellow, blue, and purple Reishi have been explored for their potential health-beneficial properties [2]. Of them, red Reishi (*G. lucidum*) and black Reishi (*G. sinensis*) have shown the most significant health-strengthening effects [3]. East Asian countries consumed Reishi for their medicinal value rather than nutritional value to extend the life span and increase youthful vigour and vitality [1].

*G. lucidum*, a cosmopolitan mushroom species, is a polypore rack mushroom that changes color during the morphogenesis process from orange-white to bright red. The major chemical constituents of *G. lucidum* and related species include polysaccharides, including triterpenoids, nucleotides, sterols, steroids, fatty acids, and proteins/peptides with the most pharmacologically active compounds triterpenoids and polysaccharides [4]. It has been reported that *G. lucidum* contains over 400 bioactive compounds, which have several medicinal effects, remarkably such as antitumor and anti-inflammatory, antimicrobial, antifungal, antiviral (specifically against herpes and HIV) effects, antioxidative, and radical scavenging effects [5–9].

Several reports revealed the successful artificial cultivation on solid substrates utilizing sawdust and agricultural wastes (rice bran, wheat bran, sugarcane bagasse, rice husks, peanut hulls, coconut fiber, banana leaves, etc.) as well as in tea waste [3]. Generally, *G lucidum* is cultivated on solid-state fermentation and takes about six months to form the fruiting body [10]. However, the process is time consuming and difficult to control, dictating other alternatives for growing the fungi. One such method of choice is the submerged cultivation of mycelia to produce bioactive compounds, which can be rapid, economically feasible, and controllable [11].

Most human diseases result from the uncontrolled production of reactive oxygen species (ROS), including free radicals. When unchecked, the endogenous mechanism for free radical scavenging in living cells can lead to insufficient neutralization of free radicals, resulting in major deterioration conditions such as cardiovascular diseases and cancer [12]. Molecules derived from natural sources such as fungi play a significant role in developing and discovering novel drugs for treatment. Currently, many active compounds from *G. lucidum* are being studied as treatment options [13].

This study cultivates the *G. lucidum* in various media and determines the optimum growth parameters (pH, temperature, and carbon sources). In addition, the determination of phenolic content, flavonoid content, antioxidant activity, and antibacterial activity of extracts from the mycelia and fruiting body was performed.

## 2. Materials and Methods

### 2.1. Strain Used

*G. lucidum* strain Philippine was purchased from the Centre for Agricultural and Technological Training, Lalitpur, and cultured in Potato Dextrose Agar (PDA) (Hi-Media, Mumbai, India) plates and slants. The cultured plates were kept at 4°C for research purposes.

### 2.2. Mass Culture

#### 2.2.1. Culture on Synthetic Logs

A synthetic log was prepared by mixing sawdust powder, raw wheat bran, and raw rice bran. Polypropylene bags (12 × 12 cm^2^) were filled with 200 g of substrate, sealed with a cotton plug, autoclaved, and maintained to 65% moisture. The prepared synthetic logs were inoculated with fresh spawn and incubated for 60 days at 28°C in diffused light with a relative humidity of 90–95%, which was maintained with sprinkling sterile water (0.1% calcium) twice daily. Few 1 mm pores were punched, and sterile cotton was loosely plugged for air circulation.

#### 2.2.2. Culture in Potato Dextrose Broth (PDB)

PDB was prepared in the laboratory by boiling potatoes (300 g) in 1 L water and filtering to obtain the potato broth. Glucose (25 g) was added, and the final pH was adjusted to 5.0 before autoclaving. The sterile media were then inoculated with 2-3 pieces of fresh mycelia taken from potato dextrose agar (PDA) plate and cultured in a shaking incubator (120 rpm) at 28°C for 18 days.

#### 2.2.3. Culture on PDA

PDA plates inoculated with fresh and actively growing mycelia and incubated at 28°C for one week to obtain fresh inoculums as well as for one month for extraction. PDA plates were maintained at 4°C for long-term storage. The growth optimization of the culture was carried out for optimal growth and further experiments.

### 2.3. Growth Parameter Optimization in Plate Culture

#### 2.3.1. pH Optimization

PDB (100 mL) was prepared and adjusted to varied pH from 3.5 to 6.75 (0.25 interval). Then, agar powder (1.5 g) was added to each bottle, shaken, and autoclaved before pouring into sterile Petri plates in triplicates. A very minute mat with agar was plugged by using a borer and inoculated into plates, incubated at 28°C for four days, and their margin was marked. An increase in mycelial length was reported every day, and the growth rate was calculated.

#### 2.3.2. Temperature Optimization

Laboratory-prepared PBD was adjusted to pH 5.0. After adding agar, the plates were incubated at different temperatures (20°C to 36°C) in triplicates and corresponding control for four days. An increase in mycelia length was measured on the subsequent days, which indicated lengthwise growth per day.

#### 2.3.3. Carbon Source Optimization

Liquid broth (LB) (100 mL) media were prepared without a carbohydrate source, and pH was adjusted to 5.0. Then, 15 g different carbohydrate sources (sucrose, trehalose, glucose, lactose, cellulose, maltose, sorbose, and xylose) as labeled in the flask were added into respective flasks and autoclaved. Each medium was poured into plates in triplicates, inoculated with small mycelium spawn, and incubated for four days at 28°C, and their margin (apex) was marked. Everyday, the increase in mycelia length was measured, which indicated lengthwise growth per day.

### 2.4. Sample Preparation and Extraction

The fruiting bodies from solid culture and upper mycelia culture from lawn culture were harvested and then dried at 60°C until a constant weight was obtained. The harvested culture was powdered using liquid nitrogen (−196°C) and crushing by using a mortar and pestle. 10 gm of each powdered sample was extracted by 400 mL ethanol using Soxhlet's apparatus at 60°C, and the extract was dried using a rotary vacuum evaporator. Finally, a 100 mg/mL solution was prepared as a working solution.

### 2.5. Phytochemical Estimation

#### 2.5.1. Total Phenolic Content Estimation

Total phenolic content was determined with the Folin–Ciocalteu (FC) reagent as in [14]. Briefly, 100 *μ*L of each sample was dissolved in 500 *μ*L (1 : 10 v/v dilution) FC reagent and 1.5 mL (20%) sodium carbonate. The mixture was vortexed for 30 s and incubated for 2 h in the dark at room temperature for color development. Absorbance was then measured at 765 nm using the Genesys UV/Vis spectrophotometer (CA, USA). Total phenolic content (TPC) was measured as milligrams of gallic acid equivalent/g (mgGAE/g) of sample dry weight using the standard curve and the equation.

#### 2.5.2. Total Flavonoid Content (TFC) Estimation

Total flavonoid content in the extracts was estimated using the aluminium chloride colorimetric method [14]. Briefly, 0.5 mL of plant extract (20 mg/mL) was mixed with 0.1 mL of 10% aluminum chloride, 0.1 mL of 1.0 M potassium acetate, and 1.3 mL 90% ethanol. Absorbance was measured at 415 nm in the Genesys UV/Vis spectrophotometer after incubating the mixture for 40 min at room temperature. TFC was then calculated using quercetin as a standard and expressed as mg quercetin equivalent/g (mgQE/g) of sample dry weight.

### 2.6. Antibacterial Activity

Six clinical strains of bacteria (*Staphylococcus aureus, Pseudomonas aeroginosa, Bacillus subtilis, Salmonella typhii, Klebsiella pneumoniae, and Klebsiella oxytoca)* were obtained from Manmohan Memorial Community Hospital, Thamel, Kathmandu. The antibacterial activity was determined by the agar well diffusion method according to the work in [15]. Briefly, MHA agar plates were inoculated with bacterial strain under aseptic conditions, and 6 mm wells were filled with 20 *μ*L of the test samples (mature fruit extract, lawn culture mycelia) and incubated at 37°C for 24 hours. The diameter of the zone of inhibition was measured in millimeters. Likewise, 18–24 h-old single bacterial colonies on agar plates were used to prepare the bacterial suspension with the turbidity of 0.5 McFarland standard (equivalent to 1.5 × 10^8^ colony-forming units (CFU)/mL). The turbidity of the bacterial suspension was measured at 600 nm. Ethanol was used as a negative standard, while penicillin and erythromycin were used as positive standards.

### 2.7. *In Vitro* Antioxidant Assay

#### 2.7.1. Free Radical Scavenging Assay (RSA)

Free RSA was measured as described in [14]. Briefly, ethanol (1 mL) containing *G. lucidum* extract in different concentrations was added to 0.4 mL of 0.2 mL DPPH (2,2-diphenyl-1-picrylhydrazyl) solution. Also, ascorbic acid (3.125 *μ*g/mL–100 *μ*g/mL) as standard was taken in different test tubes. The sample volume was adjusted to 1 mL, adding ethanol. Then, 0.4 mL DPPH was added to these tubes, shaken gently, and allowed to stand for 45 min at room temperature in the dark. Then, absorbance was measured at 517 nm in the Genesys UV Vis spectrophotometer. RSA was expressed as the inhibition percentage and calculated as follows.

Abs_control_ is the absorbance of DPPH radical in methanol, and Abs_sample_ is the absorbance of DPPH radical in sample extract. IC_50_ value, which represents the minimum inhibitory concentration of extract required to scavenge 50% of the DPPH free radicals, was calculated as

(1) $$\begin{array}{c} {\text{IC}}_{50}=\text{EXP}\left(\text{LN}\left(\text{conc}>50\%\right)-\left(\frac{\text{pi}>50\%-50}{\text{pi}>50\%-\text{pi}<50\%}*\text{LN}\left(\frac{\text{conc}>50\%}{\text{conc}<50\%}\right)\right)\right). \end{array}$$

#### 2.7.2. Ferric Reducing Antioxidant Assay (FRAP)

FRAP was performed similarly to the protocol adopted by Subhashini et al. [16]. Briefly, reducing power ability was measured by adding sample extract to 2.5 mL phosphate buffer (0.2 M, pH 6.6) and 2.5 mL potassium ferricyanide (1%, w/v) and incubated for 30 min at 50°C. Then, 2.5 mL of 10% trichloroacetic acid was added to the mixture and centrifuged for 10 min at 3000*g*. 2.5 mL of the supernatant was then diluted with 2.5 mL distilled water and mixed with 0.5 mL freshly prepared ferric chloride (0.1%, w/v). The absorbance was measured at 700 nm using the Genesys UV Vis spectrophotometer after incubating for 30 min. Increased absorbance of the reaction mixture indicated increased reducing power. All experiments were performed in triplicate using butylated hydroxytoluene as a positive control.

#### 2.7.3. Phospho-Molybdenum Method

The total antioxidant estimation of *Ganoderma* extracts was carried out according to the work in [6]. Briefly, 0.1 mL aqueous extract (100 *μ*g/mL) was mixed with 1 mL of the reagent solution (0.6 M H_2_SO_4_, 28 mM sodium phosphate, and 4 mM ammonium molybdate). Capped tubes were then incubated in a water bath at 95°C for 90 min and cooled to room temperature. Absorbance was measured at 695 nm in the Genesys UV Vis spectrophotometer. Total antioxidant activity was expressed as ascorbic acid equivalent in mg/g of the extract.

### 2.8. Statistical Analysis

All the assays were carried out in triplicates. One-way analysis of variance (ANOVA) followed by Tukey's test (*p* < 0.05) was performed to test any significant difference among means using Statistical Package for the Social Sciences (SPSS) v. 20.0 (IBM Corp., NY, USA).

## 3. Results and Discussion

### 3.1. Influence of Culture Media

The mycelia growth pattern of *G. lucidum* on three different culture media was completely different from one another.

#### 3.1.1. Growth Pattern in PDA Media

The mycelium on PDA media initially produced straight, velvety, whitish cottony intermingled fabric mat-like colonies, which turned into yellowish-brown patches with time (45 days' incubation). Fresh growing apex had straight silken microfibers heading at the top with an interfiber distance of nearly 0.5 mm.

#### 3.1.2. Growth Pattern in Synthetic Logs

Saw dust-powered supplement with raw wheat bran and raw rice bran was used as the previous investigation on the cultivation of *G. lucidum* using different biomasses which showed saw dust is a good substrate among different substrates [17]. Polypropylene bags (200 g substrate) were covered with spawn in 22 days. However, bags were opened before browning. The yellowish-brown color appeared at the top and sides in still closed bags, indicating more oxygen is necessary for sporophore formation as lignin degradation takes place only in aerobic conditions. After six days, the crown changed into a thick cap with a white margin. Concentric striations were seen on a flattened cap and deep-brown coloration at the center with its thickening. According to Sudheer et al., the shape and color of fruiting bodies are dependent on the light provided during the cultivation [9]. Once harvested, second and third fruiting bodies also developed from the synthetic log having enough substrates. To facilitate irrigation and nutrient absorption from soil for further fruiting, logs were buried in humid soil rich in humus before primordial formation.

#### 3.1.3. Growth Pattern in PDB Media

In shaking culture after 18 days, the biomass remained submerged and round wet cotton plug like bulk. In contrast, stationary culture remained floating and spread fully, forming a disc 8 mm in thickness outwardly and 4 mm inwardly with a hydrophobic upper surface and jellied underneath. Biomass was filtered, dried, and weighed about 1.08 g/100 mL from shaking culture. However, Yang et al. reported, on long incubation (45 days), the upper surface in stationary culture changed to yellowish-brown. Mycelia from the disc started to grow upward, adhering to the bottle glass wall as the substratum with almost no mycelia remaining free in the bulk liquid similar to the immobilized culture with foam sheet [16]. Moreover, the medium was shaken for four days and kept still for having the most biomass and ganoderic acid accumulation. Maturation takes about 90 days for carpophores, whereas suitable biomass and specific metabolites can be enriched in less than 20 days [18].

### 3.2. Influence of Physical Factors in Lawn Culture

A semisolid medium was used to optimize variables for the maximum mycelial growth. PDA plates were incubated at room temperature in diffused or no light and 90–95% relative humidity. Ventilation was facilitated to increase fresh air to exchange CO_2_ gas.

#### 3.2.1. pH

**Figure 1:**
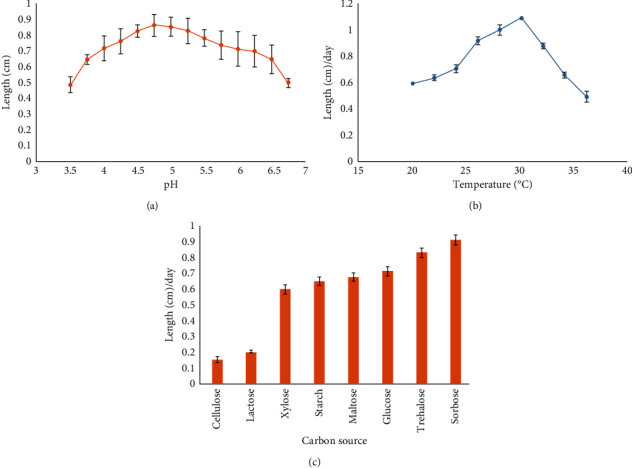
Growth optimization of *G. lucidum* using different parameters: (a) pH; (b) temperature; and (c) carbon source in PDA plates.

Many fungi can grow in acidic to slightly alkaline media, adjusting to various pH adversities within pH 2.0 and 9.0 [8]. The optimal pH range for promoting mycelia growth of *Ganoderma* species has been reported to be 5.5–6.0 [10]. However, in this research, higher growth was observed between pH 4.5 and 5.5 (Figure 1(a)). About 1.08 cm increase in mycelial length per day was observed over a pH range of 4.5 to 5.5. Higher and sustained mycelial growth was obtained in the alkaline medium than in the acidic medium, whereas growth tended to halt at or below pH 3.0. Similarly, Lee et al. showed that *G*. *lucidum* had a broad pH range of 5.0–9.0 for growth, and mostly favorable growth was found at pH 5.0 [10].

#### 3.2.2. Temperature

Generally, the minimum and maximum principal temperatures for the mycelial growth and biomass of *G. lucidum* were 9°C and 32°C, respectively [16]. Fungi can naturally adjust to various fluctuations in temperature as they can adapt to induce sporulation on adverse conditions to protect the germplasm. In the present study, the highest growth was observed between 28°C and 32°C at a previously optimized pH of 5.0 (Figure 1(b)). Lengthwise mycelial growth increased with increasing incubation temperature and peaked at 30°C, plunging downwards. Like the current study, Jayasinghe et al. reported the most suitable temperature for mycelia growth at 30°C [16].

#### 3.2.3. Carbon Source

The carbon source was optimized by using different carbohydrates in basal minimum (10 g/L). Usually, fungi prefer glucose over other carbon compounds due to fast metabolization for cellular energy production [16]. At the optimized temperature (30°C) and pH (5.0), sorbose was found to be the best carbon source for mycelial growth of *G. lucidum,* which was closely followed by trehalose. Other substrates, i.e., glucose, maltose, and starch, followed them in descending order. Both sorbose and trehalose yielded 1.08 cm of mycelial growth per day. On the contrary, *G. lucidum* was almost reluctant to utilize cellulose and lactose as carbon sources (Figure 1(c)).

### 3.3. Extraction, Phenolic, and Flavonoid Content

Extraction yields were the highest for extracts of *G. lucidum* MCE (11.000%) and then FCE (10.625%). The mycelia extracts differed significantly in their flavonoid and phenolic contents. Thus, total phenol concentrations in mature fruit and lawn mycelia were 5.6 *μ*g GAE/mg and 0.9448 *μ*g GAE/mg, while flavonoid content varied from 45.875 *μ*g QE/mg to 34.375 *μ*g QE/mg (Table 1). Some researchers have reported and quantified flavonoids in *G. lucidum* [8].

### 3.4. Bioassay

#### 3.4.1. Antibacterial Activity

**Figure 2:**
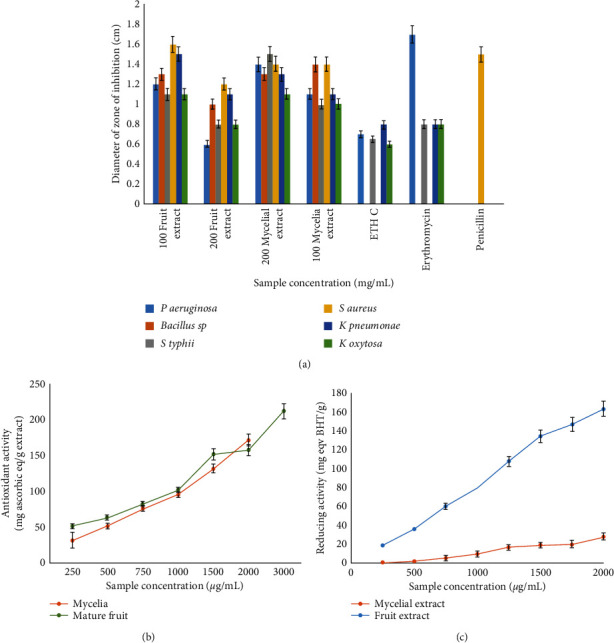
Different activities as shown by the *G. lucidum* extracts. (a) Bar graph showing inhibition zones with *G. lucidum*'s extracts on various opportunistic bacteria compared to standard antibiotics and control ethanol; (b) graph illustrating the reducing power of *G. lucidum* extracts compared to BHT as standard; BHT = butylated hydroxytoluene, Myc = mycelia, and FRT = fruit.; and (c) graph for total antioxidant potential assay by the phospho-molybdenum method; Myc = mycelia, MFRT = mature fruit, and ACRB = ascorbic acid.

The zone of inhibition in the agar well diffusion method for FCE and MCE at concentrations 200 and 100 *μ*g/mL with standard drug erythromycin (ERM) and penicillin (PNC) is presented in Figure 2. It is observed that both 200 mg/mL and 100 mg/mL inhibited all the bacteria, and the results were more promising than the corresponding control antibiotics. Antimicrobial compounds in mushrooms, such as terpenes, lectins, and polysaccharides, act on the bacterial cytoplasmic membrane [7]. For *G. lucidum,* various extracts are equally effective when compared with gentamycin sulphate. Quereshi et al. reported that the most active antimicrobial components are generally water insoluble; it is expected that low-polarity organic solvents would yield more active antimicrobial extracts, and so, ethanol is suitable to some extent [19]. According to Mishra et al., *G. lucidum* and other species extract combined with chemotherapeutic agents have been used to treat various bacterial infections [15]. Triterpenoids and polysaccharides play a key role in the antibacterial activity. However, terpenoids, isoflavonoids, and tannins have stronger antimicrobial effects than other compounds singly. Smania et al. observed methyl australate, a derivative from *G. lucidum,* showing maximum antibacterial activity against *E. coli*, *P. aeruginosa,* and *S. aureus* while the least inhibition zone was recorded for *Bacillus* species [20]. Moreover, some components such as ganomycin, triterpenoids, and aqueous extracts from *G. lucidum* species have a broad-spectrum *in vitro* antibacterial activity against Gram-positive bacteria, Gram-negative bacteria, and *Helicobacter pylori*.

### 3.5. *In Vitro* Antioxidant Assay

#### 3.5.1. DPPH Scavenging Assay

The scavenging activity profiles of the FCE and MCE are shown in Figure 2. The RSA increased with the concentrations of mature fruit extract; however, the mycelia extract was more consistent with the increasing concentration. The mature fruit extract scavenging effect on DPPH at concentrations ranging from 100 to 300 mg/mL was thrice. In particular, there was an increase in the scavenging effect of mature fruit extracts up to a 300 mg/mL concentration (60%), beyond which there was little significant increase, even up to 1000 mg/mL. On the contrary, mycelia extract exhibited a little progressive increase in the scavenging effect from 10–1000 mg/mL (about 55%).

Antioxidant capacity is expressed as their IC_50_ is calculated and compared with standards such as ascorbic acid. The lower the IC_50_, the higher will be its antioxidant potential and vice versa [14]. IC_50_ values for ascorbic acid, FCE, and MCE were found to be 30.60134 *μ*g/ml, 176.7767 *μ*g/mL, and 157.4901 *μ*g/mL, respectively. Ascorbic acid is a far more potent antioxidant than *G. lucidum* extract, having far high IC_50_ values. FCE showed a little higher IC_50_ value than MCE. Ascorbic acid is a pure compound having high antioxidant activity. Despite its lower value, *G. lucidum* extracts showing a positive trend with ascorbic acid signified potent antioxidant ability.

#### 3.5.2. Ferrous Reducing Assay (FRA)

The FRA assay is widely used to estimate the total antioxidant power of mushrooms by their capacity to convert ferricyanide (Fe^3+^) to ferrocyanide (Fe^2+^) [14]. In the present study, the fruiting body had higher reducing power than the mycelia. The average reducing power of 13.58 ± 3.18 mg eqv BHT/g and maximum only of 17.25 mg eqv BHT/g at 2 mg/mL were observed for the mycelial extract. However, the average reducing power value for the fruiting body was 104.08 ± 7.59 mg eqv BHT/g with a maximum value of 111.67 mg eqv BHT/g at 1.5 mg/mL fruit extract. In *G. lucidum*, phenolic compounds are directly correlated to their antioxidant activities [14].

#### 3.5.3. Phospho-Molybdenum Method

Among both the extracts of *G. lucidium*, fruit extract showed higher antioxidant potential (150.6 ± 56.92 mg ascorbic acid equivalent/g extract) than mycelia extract (144.28 ± 81.72 mg ascorbic acid equivalent/g extract) (Figure 2(c)). However, there was no significant difference in the activities between these extracts. Higher results indicate that both the *Ganoderma* extracts have potent radical scavenging effects for nitrous oxide and superoxide anion radicals. This property might be due to the presence of carotenoids (terpenoids) in *Ganoderma* extracts.

## 4. Conclusions

In conclusion, *G*. *lucidum* can be optimally cultivated in sawdust substrate and PDA plates. The optimum conditions for growth were temperature 28–32°C, pH 4.5–5.5, carbon source sorbose, and trehalose. A similar yield percentage was obtained from both mycelia and fruiting bodies as 10.62% and 11%, respectively. The phytochemical properties, antibacterial properties, and antioxidants were comparable in both *G. lucidum* mycelia grown in the lab and sawdust substrate logs. Thus, further evaluation and identification of specific compounds are required to fully understand the mechanism in the biological system and drug discovery.

## Figures and Tables

**Table 1:** Comparative yield and phytochemical composition of the mature fruit and mycelia of *Ganoderma lucidum* strain Philippine.

S. no.	Raw material	Crude weight (g)	Extract yield (g)	Yield (%)	Phenolic content (mg GAE/gm of extract) ± SD	Flavonoid content (mg QE/gm of extract) ± SD
1	Mature fruit	8	0.85	10.625	5.6 ± 0.080	45.875 ± 0.060
2	Lawn mycelia	5	0.55	11	0.9448 ± 0.010	34.375 ± 0.020

## Data Availability

Data used to support this study are included in the article.
